# Accurate Identification of Subclones in Tumor Genomes

**DOI:** 10.1093/molbev/msac136

**Published:** 2022-06-24

**Authors:** Navid Ahmadinejad, Shayna Troftgruben, Junwen Wang, Pramod B Chandrashekar, Valentin Dinu, Carlo Maley, Li Liu

**Affiliations:** College of Health Solutions, Arizona State University, Phoenix, AZ 85054, USA; Biodesign Institute, Arizona State University, Tempe, AZ 85281, USA; College of Health Solutions, Arizona State University, Phoenix, AZ 85054, USA; College of Health Solutions, Arizona State University, Phoenix, AZ 85054, USA; Department of Health Sciences Research and Center for Individualized Medicine, Mayo Clinic Arizona, Scottsdale, AZ 85259, USA; College of Health Solutions, Arizona State University, Phoenix, AZ 85054, USA; Biodesign Institute, Arizona State University, Tempe, AZ 85281, USA; College of Health Solutions, Arizona State University, Phoenix, AZ 85054, USA; Biodesign Institute, Arizona State University, Tempe, AZ 85281, USA; Biodesign Institute, Arizona State University, Tempe, AZ 85281, USA; College of Health Solutions, Arizona State University, Phoenix, AZ 85054, USA; Biodesign Institute, Arizona State University, Tempe, AZ 85281, USA

**Keywords:** cancer evolution, genomics, statistical modeling

## Abstract

Understanding intratumor heterogeneity is critical for studying tumorigenesis and designing personalized treatments. To decompose the mixed cell population in a tumor, subclones are inferred computationally based on variant allele frequency (VAF) from bulk sequencing data. In this study, we showed that sequencing depth, mean VAF, and variance of VAF of a subclone are confounded. Without considering this effect, current methods require deep-sequencing data (>300× depth) to reliably infer subclones. Here, we present a novel algorithm that incorporates depth-variance and mean-variance dependencies in a clustering error model and successfully identifies subclones in tumors sequenced at depths of as low as 30×. We implemented the algorithm as a model-based adaptive grouping of subclones (MAGOS) method. Analyses of computer simulated data and empirical sequencing data showed that MAGOS outperformed existing methods on minimum sequencing depth, decomposition accuracy, and computation efficiency. The most prominent improvements were observed in analyzing tumors sequenced at depths between 30× and 200×, whereas the performance was comparable between MAGOS and existing methods on deeply sequenced tumors. MAGOS supports analysis of single-nucleotide variants and copy number variants from a single sample or multiple samples of a tumor. We applied MAGOS to whole-exome data of late-stage liver cancers and discovered that high subclone count in a tumor was a significant risk factor of poor prognosis. Lastly, our analysis suggested that sequencing multiple samples of the same tumor at standard depth is more cost-effective and robust for subclone characterization than deep sequencing a single sample. MAGOS is available at github (https://github.com/liliulab/magos).

## Introduction

The development of a tumor is an evolutionary process that typically initiates from a single clone and grows into a diverse population of cells via incessant mutations and selections (Nowell 1976; Greaves and Maley 2012; Miura et al. 2018). As a tumor progresses over time and space, different cell populations (i.e., subclones) emerge, expand, and diminish, leading to a heterogeneous neoplasm. Studies have revealed that distinctive subclones are involved in metastatic spread, drug resistance, and other clinically important characteristics of cancers (Jamal-Hanjani et al. 2017; McGranahan and Swanton 2017). Understanding intratumor heterogeneity and evolutionary dynamics provides valuable insights into disease mechanism and clinical management of cancers (Gerlinger and Swanton 2010; Aktipis et al. 2011; Ma et al. 2012; Nik-Zainal et al. 2012; Fisher et al. 2013; Andor et al. 2014).

Whole-exome sequencing (WES) and whole-genome sequencing (WGS) are common approaches to examine intratumor heterogeneity (Egan et al. 2012; Landau et al. 2013; Schwartz and Schaffer 2017). By tracking relative abundances of genomic variants in a collection of cancerous cells, scientists aim to quantify the genetic diversity of a tumor and to reconstruct the subclonal compositions and phylogenies. As single-cell genome sequencing is a promising technology to examine genetic profiles of individual cells, uneven genome coverage, low accuracy of variant calls, and prohibitive cost currently limit its usage in subclonal investigations (Navin et al. 2010, 2011; Hughes et al. 2014; Gawad et al. 2016). Current studies and likely many others in the future still rely on bulk sequencing to interrogate mixed tumor cells, then apply computational approaches to deconvolute the cell population into clones and subclones.

SciClone (Miller et al. 2014), PyClone (Roth et al. 2014), and Expands (Andor et al. 2014) are popular computational methods used to infer subclones from bulk sequencing data. These methods share a common framework, in which variant allele frequency (VAF; i.e., fraction of reads containing a specific mutant allele among all reads covering the locus) serves as a surrogate of cellular prevalence (i.e., fraction of cells carrying this mutant among all cells). Decomposing a mixed cell population is then formulated as a clustering problem such that variants with similar VAF are grouped together representing a clone or a subclone (Miller et al. 2014). Cluster analysis seeks solutions that minimize within-cluster variance and maximize between-cluster variance (Saxena et al. 2017). However, in bulk sequencing data, within-cluster variance of VAF changes with sequencing depth. In a study that sequenced the same tumor sample at different depths, the inferred clusters became more dispersed as sequencing depth decreased (Griffith et al. 2015). Furthermore, a cluster with a high mean VAF tends to be more dispersed than a cluster with a low mean VAF does. Such depth-variance dependence and mean-variance dependence are present in many subclonal analysis results (Griffith et al. 2015; Deveau et al. 2018; Chkhaidze et al. 2019). Surprisingly, current methods overlook these confounders, leading to unexplained variance that may reduce decomposition accuracy, especially when within-cluster variance and between-cluster variance lack a strong contrast. To curtail the adverse impact, current methods require a minimum sequencing depth of 100×. An independent evaluation of SciClone further showed that consistent subclonal characterization can only be achieved when sequencing depth exceeds 300×. This precludes analysis of an overwhelming majority of tumors sequenced to date, including those from collaborative consortia, such as the TCGA project (average depth = 68^×^; Grossman et al. 2016). New methods capable of accurately identifying subclones without the needs for deep-sequencing data will help discover information hidden in the myriad of established genomic data that are currently unexploited.

In this study, we first examine the statistical significance and the magnitude of depth-variance dependence and mean-variance dependence in different scenarios. We then present a model-based adaptive grouping of subclones (MAGOS) method that incorporates these confounders in an error model at the subclonal level. We show that MAGOS accurately identifies subclones in tumors sequenced at depths as low as 30×. MAGOS also achieved an acceleration of 4- to 120-fold in processing time when compared with SciClone and PyClone. We implemented MAGOS as an R package that is freely available at github (https://github.com/liliulab/magos).

## Results

### Variance of VAF Decreases with Sequencing Depth and Increases with Mean VAF

To examine the depth-variance dependence and mean-variance dependence in empirical data, we analyzed a tumor genome that was sequenced at three different depths (Griffith et al. 2015). This data set contains 1,343 high-quality validated somatic single-nucleotide variants (SNVs) in a primary acute myeloid leukemia. Three sequencing experiments, namely “Illumina capture,” “Illumina WGS,” and “Ion Torrent capture” produced reads supporting these SNVs at a median depth of 1,394×, 326×, and 42×, respectively. Griffith et al. combined reads from these experiments and performed SciClone analysis, which identified six clusters with size ranging from 43 to 911 SNVs and mean VAF ranging from 0.001 to 0.47. Because true subclones in this tumor are unknown, they treated these clusters as benchmark and mapped data from each experiment to these clusters.

**Fig. 1.**
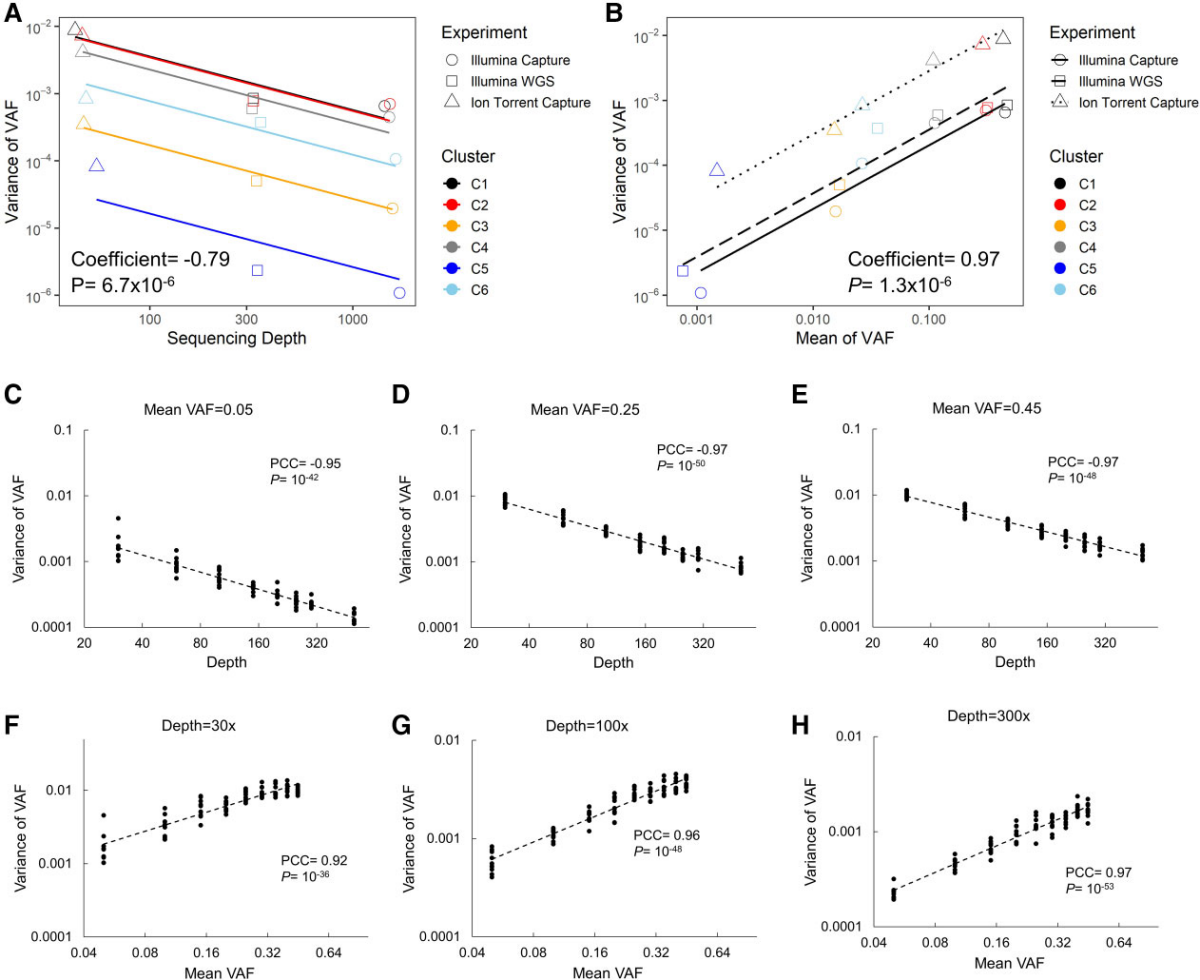
Variance of VAF decreases with sequencing depth and increases with mean VAF. (*A*, *B*) Griffith et al. (2015) conducted three experiments to sequence genomic variants in a tumor at different depths and grouped the variants into six clusters. In this data set, the variance of VAF in a cluster is negatively associated with sequencing depth (*A*) and positively associated with mean of VAF (*B*). Fitted lines represent mixed effect models in which sequencing depth and mean VAF have fixed effect (slope) and clusters have random effect (intercept) on variance of VAF. Coefficient and *P*-value of fixed effect are displayed. (*C*–*H*) Given a target sequencing depth and a target mean VAF, ten clusters were simulated computationally. When mean VAF is fixed, variance of VAF is negatively correlated with sequencing depths (*C*–*E*). When sequencing depth is fixed, variance of VAF is positively correlated with mean VAF (*F*–*H*). Pearson correlation coefficient (PCC) and *P*-value are displayed.

Using these data, we built a mixed-effects regression model in which sequencing depth and mean VAF had fixed effect on within-cluster variance of VAF and cluster had a random effect (details in Materials and Methods section). This model estimated that within-cluster variance of VAF was negatively associated with sequencing depth (coefficient = –0.79, *P* = 6.7 × 10^−6^; fig. 1*A*), and positively associated with mean VAF (coefficient = 0.97, *P* = 1.3 × 10^−6^; fig. 1*B*).

To ensure the detected associations were not artifacts of the different sequencing platforms used in the three experiments, we performed computer simulations (details in Materials and Methods). Based on Poisson distribution, we down-sampled reads in the Illumina capture experiment to a target sequencing depth and a target VAF, forming a simulated cluster. We examined 8 levels of sequencing depths ranging from 30× to 500×, and 9 levels of mean VAF ranging from 0.05 to 0.45, producing 72 combinations. Correlation tests using the simulated data confirmed the depth-variance dependence (Pearson correlation coefficient PCC min = –0.97, max = –0.95, all *P* < 10^−42^; fig. 1*C*–*E*) and mean-variance dependence (PCC min = 0.92, max = 0.97, all *P* < 10^−36^; fig. 1*F*–*H*).

### New Method: MAGOS

The significant associations between variance of VAF and sequencing depth and mean VAF suggested the confounding effects were not negligible. Our new method MAGOS explicitly models these dependencies in cluster analysis. The input to MAGOS contains read counts supporting reference alleles and mutant alleles of each somatic variant in a tumor. The output contains cluster assignments and confidence scores. MAGOS supports SNVs and copy number variants (CNVs) obtained from one or more samples, and simultaneously estimates sample purities. Below we first present the algorithm for a single-tumor sample and then generalize it to multiple samples. Because an inferred cluster corresponds to a subclonal expansion, we use clusters and subclones interchangeably.

Given a set of diploid SNVs in a tumor sample, our task is to find clusters of SNVs with similar VAF. For an SNV*i*, we denote the total number of reads aligned to this position as sequencing depth *e*_*i*_, and denote the fraction of reads containing the mutant allele as VAF *v*_*i*_ ∈ (0, 1). If a variant has a heterozygous genotype in germline and the VAF is >0.5, we swap the reference and mutant alleles and adjust the *v*_*i*_ accordingly. For a set of *m* SNVs belonging to the same cluster, we model their VAF using a beta distribution Beta(*α*, *β*) with shape parameters *α* and *β*. In a beta distribution, *α* + *β* approximates the sample size, which is equivalent to the mean sequencing depth $\bar{e}$; and $\frac{\alpha }{\alpha +\beta }$ approximates the expected frequency, which is equivalent to mean VAF $\bar{v}$. Therefore, we derive

(1) $$\{\begin{array}{c} \alpha +\beta =\bar{e}\\ \frac{\alpha }{\alpha +\beta }=\bar{v} \end{array}\;\;$$

**Fig. 2.**
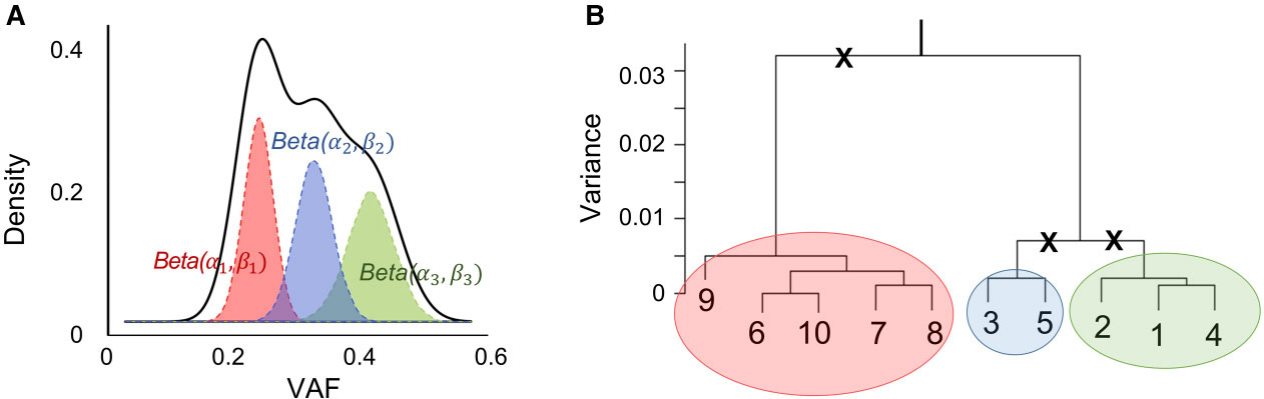
MAGOS algorithm. (*A*) Beta mixture distribution. The observed distribution (solid unshaded curve) of VAF is a combination of multiple hidden groups of VAFs, each forming a beta distribution (dashed shaded curves) defined by different parameters. (*B*) Hierarchical clustering and adaptive partitioning. In this example, ten variants at the leaf nodes are progressively grouped into clusters to form a tree structure. To partition the tree, MAGOS follows the root-to-leave paths. At each branching point, the variance of VAF of the clade is compared with the expected variance. A cluster is accepted if the variance is lower than or equal to the expected value. Otherwise, it is rejected (marked by crosses), and partitioning continues. In this example, three clusters (shaded circles) are accepted.

Based on equation (1), the variance of VAF is $\mathrm{var}(v)=\frac{\alpha \beta }{{\left(\alpha +\beta \right)}^{2}(\alpha +\beta +1)}=\frac{\bar{v}(1-\bar{v})}{\bar{e}+1}$. As $\bar{v}$ is constrained within the range of 0 and 0.5, var(*v*) is positively correlated with the mean VAF and negatively correlated with the mean sequencing depth, which reflects the observed dependencies. When multiple subclones are present, the observed VAFs are a mixture of samples from multiple beta distributions, each defined by a set of shape parameters. Therefore, identification of subclones is equivalent to decomposing mixed beta distributions, with equation (1) fit to each subclone (fig. 2*A*). We solve this problem with a two-phase algorithm that performs agglomerative hierarchical clustering and adaptive partitioning.

In the first phase, we organize SNVs into a hierarchical tree structure by progressively grouping variants with similar VAF into a cluster. Starting with leaf nodes each consisting of an individual variant, we iteratively merge a pair of nodes that has the shortest distance among all pairs to create a new cluster till all variants are merged into one root cluster. Given two nodes *C*_1_ and *C*_2_ consisting of *m*_1_ and *m*_2_ variants, respectively, we define their distance *d* as the weighted negative log-likelihood that VAF of all variants in *C*_1_ and *C*_2_ are drawn from the same beta distribution,

(2) $$d(C_{1},\;\;C_{2})=w\;\underset{i\in \{C_{1},\;C_{2}\}}{\sum }-\log(P(v_{i}\;;\;\mathrm{Beta}(\alpha ,\;\beta )))$$

where *α* and *β* are calculated by solving equation (1) and the weight $w=\frac{1}{\left(m_{1}+m_{2})\cdot \mathrm{var}(v)\cdot \mathrm{range}(v\right)}$. We compute the likelihood *P* using the density function of beta distribution that may produce values >1, leading to negative distance. Because negative distances are shorter than positive distances, negative-distance clusters will be merged before positive-distance clusters. The weight *w* has three parts. The first part, 1/(*m*_1_ + *m*_2_), is to compute the average value of log-likelihood. The second part, 1/var(*v*), is to adjust for outliers due to uneven sequencing depth. If a subclone consists of variants from genomic regions with drastically different local sequencing depths, those sequenced at low depths tend to have inflated VAFs. The mean log-likelihood is sensitive to these outliers, biased toward longer distance. Because low depth and high VAF both increase variance of VAF (fig. 1), we use var(*v*) in the weight to counteract with this effect. The third part, 1/range(*v*), also serves to adjust for these outliers that inflate the range of VAFs in the same subclone.

In the second phase, we identify boundaries of distinct beta distributions by traversing and partitioning the tree into clades (i.e., aggregation of nodes below a branching point). Unlike traditional approaches that cut the tree at a fixed branch level, we perform an adaptive partitioning (fig. 2*B*). Along the root-to-leaves path, we examine the clade at each branching point and test the null hypothesis that all VAFs in this clade are drawn from the same beta distribution. This is done by comparing the observed variance of VAF with the expected variance of VAF. Specifically, given a clade containing *m* variants, we assume they all belong to the same cluster and compute *α* and *β* by solving equation (1). We then draw *m* random samples $x_{1:m}∼\mathrm{Beta}(\alpha ,\beta )$ and calculate var(*x*). By repeating this process 1,000 times, we derive 1,000 var(*x*) values representing the null distribution. Using one-sample one-sided *t*-test, we evaluate if $\mathrm{var}(v)\;\leq \bar{\mathrm{var}(x)}$. We reject the null hypothesis if the *P*-value <0.01, which indicates VAFs of this clade are from heterogeneous beta distributions and need to be partitioned further. Otherwise, we consider this clade as homogeneous and stop traversing below this branching point. We repeat this process till we find homogeneous clades along all branches, or we reach the leaf nodes. Each homogeneous clade represents a unique cluster. For each unique cluster, we derive the corresponding beta distribution using equation (1). The confidence score of assigning a variant to a cluster is the probability that its VAF is drawn from the corresponding beta distribution. Specifically, if the VAF of the variant is lower than the mean VAF of a given cluster, the confidence score equals to the left tail probability. Otherwise, the right tail probability is taken.

When multiple samples of a tumor are available, the same SNV may have different VAFs and sequencing depths in different samples. However, because SNVs from the same subclone evolve together, changes of their VAFs shall be concordant across all samples. We then extend equation (1) to

(3) $$\{\begin{array}{c} \alpha^{s}+\beta^{s}=\bar{e^{s}}\\ \frac{\alpha^{s}}{\alpha^{s}+\beta^{s}}=\bar{v^{s}} \end{array}$$

where $\bar{e^{s}}$ is the mean sequencing depth and $\bar{v^{s}}$ is the mean VAF of these variants in sample *s*, and *α*^*s*^ and *β*^*s*^ are the two shape parameters of a beta distribution specific to this sample. To determine the between-cluster distance in each tumor sample, we extend equation (2) to

(4) $$d^{s}(C_{1},\;\;C_{2})=w^{s}\;\underset{i\in \{C_{1},\;C_{2}\}}{\sum }-\log(P(v_{i}^{s}\;;\;\mathrm{Beta}(\alpha^{s},\beta^{s})))$$

where weight *w*^*s*^ is computed for each sample *s* as 1/(*m*_1_ + *m*_2_) · var(*v*^*s*^) · range(*v*^*s*^). We then define the between-cluster distance across all *S* samples as

(5) $$d(C_{1},\;\;C_{2})=\max(d^{1},\ldots ,d^{s})$$

Thus, variants with concordant VAFs across all samples will be merged prior to variants with discordant VAFs. During tree partitioning, we evaluate if $\mathrm{var}(v^{s})\;\leq \;\bar{\mathrm{var}(x^{s})}$ for each sample *s*. We accept a clade as a single cluster if no sample produces a *P*-value <0.01.

Because the above analyses are performed on diploid SNVs not affected by CNVs, the mean VAF $\bar{v_{c}^{s}}$ of variants in the cluster *c* from the sample *s* is linearly correlated with the cellular prevalence $\rho_{c}^{s}=2\bar{v_{c}^{s}}$. In a tumor sample that is 100% pure, the cluster with mean VAF = 0.5 consists of heterozygous SNVs in the founding clone *fc*. If a tumor sample is contaminated with normal cells, the mean VAF of the founding clone ${\bar{v}}_{\;fc}$ will deviate from 0.5, and the difference is proportional to the fraction of normal cells in the admixture. Therefore, sample purity $r^{s}=\rho_{\;fc}^{s}=2\bar{v_{\;fc}^{s}}$, where *fc* corresponds to the cluster with the mean VAF closest to 0.5 in the sample *s*. Other clusters with lower mean VAF emerge at various time points after the founding clone expansion.

Lastly, we solve subclones containing CNVs. We assume that all subclones have sufficient diploid SNVs to be recognized in the previous steps, and CNVs do not introduce new clusters. Our task is to assign SNVs at CNV-affected loci to existing clusters. Because an SNV and its harboring CNV region may not belong to the same cluster, we infer their cluster assignments concurrently. Given an SNV *i* in a CNV region in sample *s*, its expected VAF is

(6) $$\hat{v_{i}^{s}}=\frac{k^{s}2\bar{v_{g}^{s}}}{\varphi_{i}^{s}}$$

where $\varphi_{i}^{s}$ is the average ploidy, *k*^*s*^ is the unknown number of copies carrying the mutant allele, and $\bar{v_{g}^{s}}$ is the mean VAF of cluster *g* this SNV belongs to. To find the most likely cluster from among existing SNV clusters {1, …, *C*}, we solve

(7) $$\underset{k^{s},\;\;g}{\arg\;\min}\;|v_{i}^{s}-\hat{v_{i}^{s}}|=\underset{k^{s},\;\;g}{\arg\;\min}\;\left|v_{i}^{s}-\frac{k^{s}2\bar{v_{g}^{s}}}{\varphi_{i}^{s}}\right|$$

where $v_{i}^{s}$ is the observed VAF. We limit the search space of *k*^*s*^ to integers between 1 and a user-defined upper limit (default = 10). Because all SNVs in the same CNV region share the same average ploidy $\varphi_{i}^{s}=2(1+\bar{v_{\omega }^{s}}(N-2))$, where *N* is the reported total copy number and *ω* is the cluster the CNV belongs to, we can infer *ω* via a grid search among all existing clusters to minimize the sum of equation (7) over all affected SNVs. Solutions to these optimization problems will produce cluster assignments of CNVs, cluster assignments of SNVs, and the number of copies carrying the mutant allele of each SNV.

### Performance on Simulated Single-Tumor Samples

We evaluated MAGOS, PyClone, and SciClone on the minimum sequencing depth and the minimum difference of mean VAF $\left(\Delta \bar{v}\right)$ between subclones that can be decomposed. Because true subclonal structures of a real tumor are unknown, we performed computer simulations (details in Materials and Methods section). Each simulated tumor contained 90% cancerous cells and 10% normal cells. We generated read counts of variants belonging to two subclones. The sequencing depth $\bar{e}$ varied across 8 levels in the range of 30× and 500×, and the mean VAF $\bar{v}$ of each subclone varied across 9 levels in the range of 0.05 and 0.45, producing 72 combinations. Within a tumor, the subclone with the high $\bar{v}$ mimicked a founding subclone, and the one with the low $\bar{v}$ mimicked a descendent subclone. For each combination of $\bar{e}$ and $\bar{v}$, we simulated ten tumors. To quantify decomposition accuracies, we computed a *J*-score based on clustering precision and recall (Ahmadinejad and Liu 2021; supplementary materials, Supplementary material online). A *J*-score takes a value between 0 and 1, with 1 indicating a perfect match between true cluster compositions and inferred compositions.

**Fig. 3.**
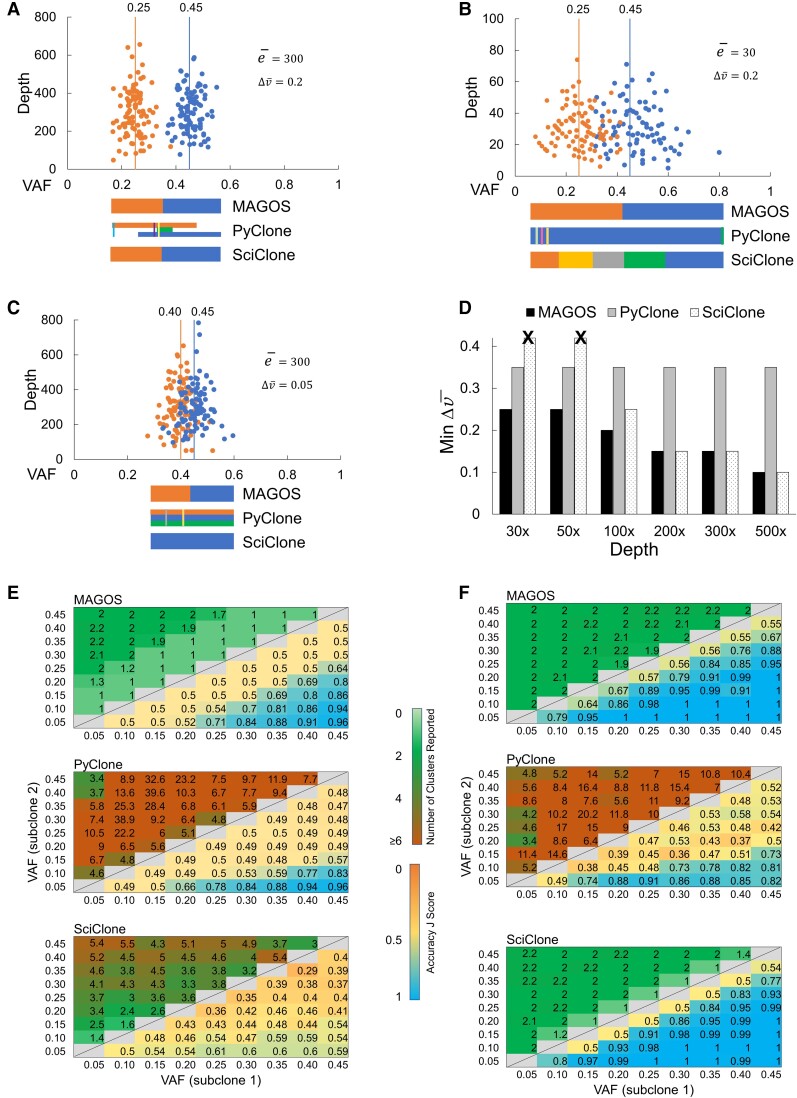
Performance of MAGOS, PyClone, and SciClone on simulated single-tumor samples, each consisting of two subclones. (*A–C*) Scatter plots of variants belonging to two simulated subclones (orange dots and blue dots). Vertical lines and the numbers above represent mean VAF of a subclone. Colored bars below a scatter plot represent clusters identified by each method. Stacked colored bars represent clusters with overlapping VAF ranges. Thin colored bars inside wide colored bars represent small clusters nested in large clusters. (*D*) Minimum ΔVAF of two subclones that can be decomposed with an accuracy *J*-score ≥0.80 by each method. Bars with a black cross above indicate *J*-scores ≥0.80 cannot be achieved. (*E*, *F*) Numbers of reported clusters (upper triangles) and *J*-scores (lower triangles) at sequencing depths of 30× (*E*) and 300× (*F*). Displayed values are averages of ten simulations. Perfect decompositions shall report two clusters and a *J*-score value of 1.

We first examined simulated tumors in which $\bar{v}$ of the founding subclone was fixed at 0.45 and $\bar{v}$ of the descendent subclone varied from 0.05 to 0.40. We presented three examples to illustrate decomposition results of MAGOS, PyClone, and SciClone. In the first example, a tumor with $\Delta \bar{v}=0.2$ and $\bar{e}=300\times$ was simulated. The founding and descendent subclones in this tumor had well-separated VAF distributions (fig. 3*A*). MAGOS and SciClone inferred two clusters correctly and had *J*-scores of 0.99. Interestingly, PyClone split each subclone into multiple clusters, many of which contained only a few SNVs, giving a *J*-score of 0.48. Adjusting algorithm parameters of PyClone did not improve the performance. However, this is not surprising because poor performance in single-sample analysis is a known limitation of PyClone (Roth et al. 2014). In the second example, we dropped the sequencing depth to 30× while keeping $\Delta \bar{v}=0.2$. VAFs in the two subclones overlapped substantially and spanned a wide spectrum from 0.09 to 0.85 (fig. 3*B*). MAGOS was still able to infer the correct number of clusters. However, misassignment of overlapping variants to the opposite cluster brought down the *J*-score to 0.66. SciClone produced excessive clusters, mistaking the large variance of VAF associated with low sequencing depth as variance caused by mixed cell populations, as expected for algorithms overlooking the depth-variance dependence. Consequently, it had a low *J*-score of 0.42. PyClone reported one cluster spanning the entire VAF range with several interspersed small clusters, giving a *J*-score of 0.40. In the third example, we reduced $\Delta \bar{v}$ to 0.05. Although the sequencing depth was kept high at 300×, it was extremely challenging to separate the two subclones (fig. 3*C*). In this case, MAGOS was the only method that recognized the existence of two clusters, although all three methods produced poor *J*-scores (MAGOS: 0.60, SciClone: 0.50, PyClone: 0.57).

Using *J* ≥0.80 as the accuracy threshold, we recorded the minimum $\Delta \bar{v}$ between two subclones at a given sequencing depth for each method. The advantage of MAGOS was the most prominent at the depths of 30× to 50× (fig. 3*D*). In these simulations, MAGOS could produce accurate decompositions with $\Delta \bar{v}$ as low as 0.25. PyClone required a $\Delta \bar{v}$ of at least 0.35. SciClone could not achieve *J* ≥ 0.80 at any level of $\Delta \bar{v}$, which is consistent with its requirement of a minimum 100× sequencing depth. MAGOS remained at the leading position until the sequencing depth increased to 200×, beyond which MAGOS and SciClone could decompose the admixtures equally well. The minimum $\Delta \bar{v}$ for PyClone remained at 0.35 across all sequencing depths, reflecting its known limitation in analyzing single-tumor samples.

Next, we examined the decomposition accuracies of all tumors without restricting the $\bar{v}$ of the founding subclone. The *J*-score of all three methods was positively correlated with the $\Delta \bar{v}$ value (linear regression coefficients for MAGOS, PyClone, and SciClone = 1.30, 1.03, and 0.87, respectively, all *P* < 10^−12^). The *J*-score was positively correlated with the sequencing depth for MAGOS and SciClone (coefficients are 0.08 and 0.15, respectively, *P* < 10^−16^), but not for PyClone (coefficient = 0.006, *P* = 0.51). At the 30× depth, MAGOS could achieve an average *J*-score ≥0.80 when $\Delta \bar{v}$ ≥0.25 (fig. 3*E*). In a total of 100 such tumors, the average *J*-score of MAGOS was 0.87, which was significantly better than that of PyClone (0.74, *t*-test *P* = 0.008) and SciClone (0.54, *P* = 3.5 × 10^−8^). As the depth increased to 300×, MAGOS could achieve an average *J*-score ≥0.80 when $\Delta \bar{v}$ ≥0.15 (fig. 3*F*). In a total of 210 such tumors, the average *J*-score of MAGOS was 0.97, which was significantly better than that of PyClone (0.65, *t*-test *P* = 2 × 10^−8^) but slightly worse than SciClone (0.99, *P* = 0.02).

MAGOS reports the probability of a variant belonging to each cluster. Variants close to cluster borders tend to have lower probability score than those close to cluster centers (supplementary material fig. S1, Supplementary material online). Low probability of assigning a variant to a cluster or similar probabilities of assigning it to different clusters warrant manual examination. For example, all the misassigned variants in fig. 3*B* had probability <0.5. SciClone also reports probability scores. However, the probability scores of misassigned variants were still high in SciClone analysis (73.2% have probability >0.9).

### Performance on Simulated Multiple-Tumor Samples

To simulate subclonal structures embedded in multiple samples from an individual tumor, we used an established method that imposes the order of subclonal expansions (El-Kebir et al. 2015). Each simulated tumor contained 200 variants distributed among three subclones. The $\bar{v}$ of each subclone was randomly selected in the range of 0–0.5. We simulated read counts from two, three, and four samples of a tumor at depths of 30×, 50×, 100×, and 300×. For each combination of sample numbers and sequencing depths, we created 40 simulations.

**Fig. 4.**
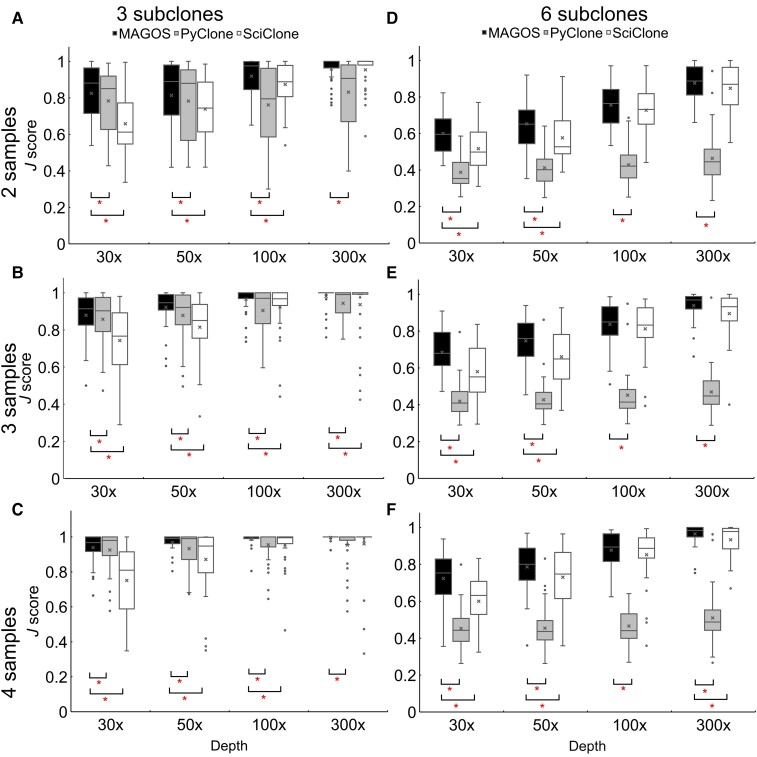
Performance on simulated multiple-tumor samples. Three subclones (*A*–*C*) and six subclones (*D***–***F*) were simulated. For each tumor, two samples (*A*, *D*), three samples (*B*, *E*), or four samples (*C*, *F*) sequenced at various depth were simulated. At a given sequencing depth, boxplots of *J*-scores from 40 simulations are presented. Asterisks indicate a significant better performance of MAGOS when compared with the other two methods.

Overall, sequencing additional samples from the same tumor and increasing the depth improved the accuracies of all three methods (multivariate linear regressions *P* < 0.002, fig. 4*A*–*C*). The performance of SciClone was more sensitive to sequencing depths than to sample numbers (*R*^2^ = 0.17 vs. 0.03). Conversely, the performance of PyClone was more sensitive to sample numbers than to sequencing depths (*R*^2^ = 0.16 vs. 0.02). The influences of these two factors on MAGOS were similar (*R*^2^ = 0.09 vs. 0.12).

In all combinations of sequencing depths and sample numbers, MAGOS consistently outperformed PyClone (all paired *t*-tests *P* ≤ 0.05). We observed the largest improvement of MAGOS over PyClone when two samples of a tumor were sequenced at the depth of 100× (mean *J*-score = 0.92 vs. 0.76). Compared with SciClone, the advantages of MAGOS were the most prominent when the sequencing depths were 30×. In these cases, MAGOS had a mean *J*-score of 0.82, 0.88, and 0.94 when two, three, or four samples of a tumor were sequenced, respectively, whereas the corresponding mean *J*-scores of SciClone were 0.66, 0.74 and 0.75 (all paired *t*-tests *P* < 10^−6^). MAGOS maintained its lead over SciClone until the sequencing depths reached 300×, where both methods achieved average *J*-scores >0.95.

We also simulated cases in which six subclones were present in a tumor. All three methods showed decreased performance when compared with three subclone simulations. However, MAGOS still outperformed the other two methods consistently (fig. 4*D*–*F*). The most significant advantage of MAGOS was with 30×–50× sequencing depth. For two samples sequenced at 100× and 300× depths, the performance of SciClone improved to the same level of MAGOS. But when more samples were sequenced, MAGOS regained its lead. PyClone had the most difficulties when six subclones were present in a tumor.

### Computational Efficiency

**Fig. 5.**
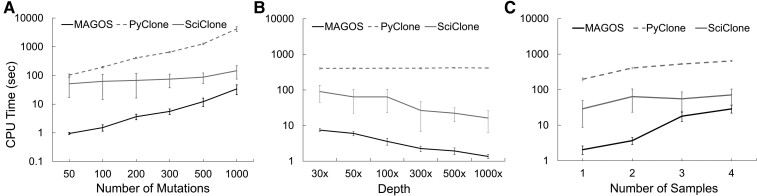
Computational efficiency of analyzing simulated data. Each method is executed as a single-threaded process on a desktop. Average CPU time over ten simulations is displayed with error bars indicating standard deviations. (*A*) Each tumor has two samples, and each sample contains 50 to 1,000 mutations sequenced at 100× depth. (*B*) Each tumor has two samples, and each sample contains 200 mutations sequenced at depths ranging from 30× to 1,000×. (*C*) Each tumor has 1 to 4 samples, and each sample contains 200 mutations sequenced at 100× depth.

The computational efficiency of MAGOS was the best among the three methods. Executed as a single-threaded process on a desktop, MAGOS took an average of 0.9 s to analyze 50 SNVs from two samples of a tumor, which was 54 times faster than SciClone (51.4 s, *t*-test *P* = 0.001) and 109 times faster than PyClone (102.8 s, *P* = 10^−10^). As the number of SNVs increased, the acceleration of MAGOS over PyClone was relatively stable at about 120×, as the acceleration over SciClone narrowed (fig. 5*A*). When the number of SNVs reached 1,000, PyClone took more than an hour to complete the analysis, SciClone took 2.4 min, and MAGOS took <0.6 min. Increasing sequencing depths helped speeding up MAGOS and SciClone but did not affect PyClone (fig. 5*B*). As expected, analyzing more samples took longer time (fig. 5*C*). Among all scenarios we tested, MAGOS was significantly faster than the other two methods (*P* < 0.05).

### Performance on Empirical Sequencing Data

The Griffith et al. (2015) study sequenced the relapsed tumor in addition to the primary tumor from the same patient. The median sequencing depth of the relapse tumor was 270×, 41×, and 47× in the Illumina capture, Illumina WGS, and Ion Torrent capture experiments, respectively. Griffith et al. combined reads from these experiments, analyzed the 1,343 high-quality SNVs in this primary-relapsed tumor pair with SciClone, and identified six clusters. Because true subclones are known, we assessed the performance of MAGOS, PyClone, and SciClone on reproducing the six benchmark clusters.

**Fig. 6.**
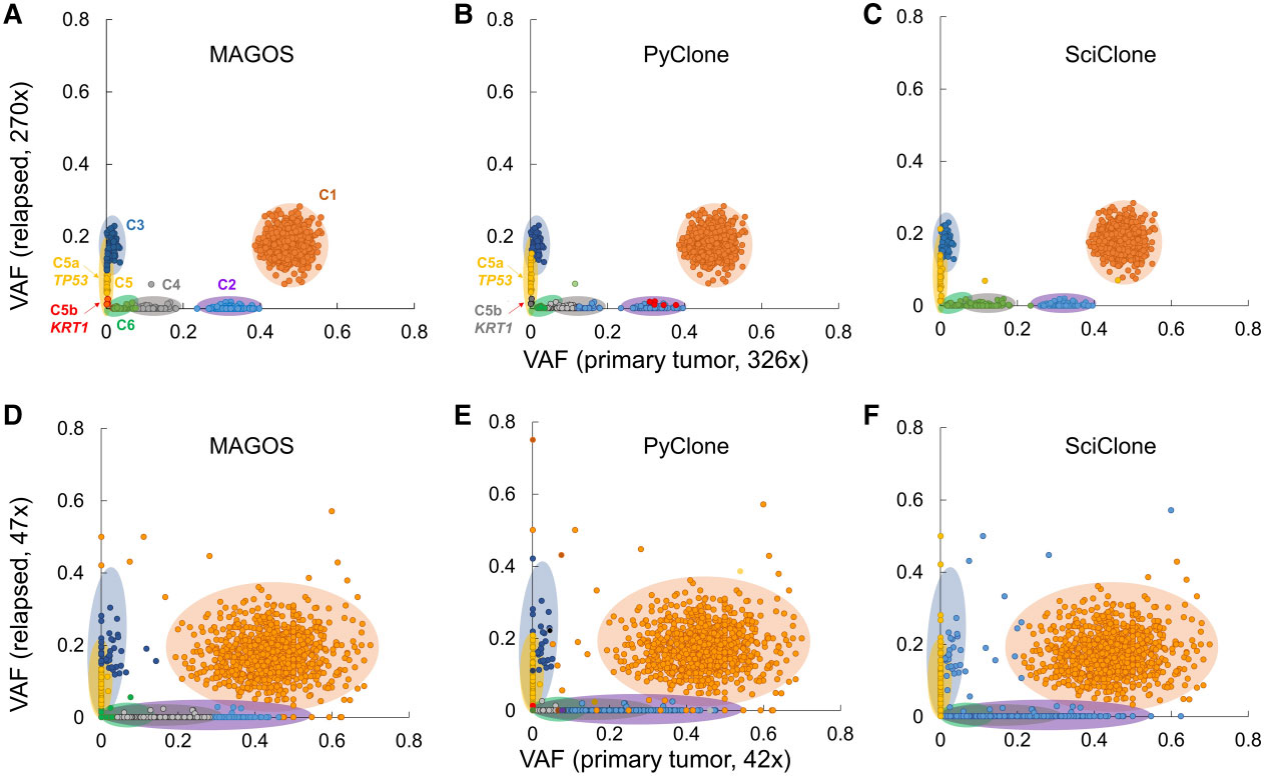
Performance on analyzing empirical sequencing data of two samples (primary tumor and relapsed tumor) from the same patient. (*A*–*C*) Scatter plots show VAFs of variants from deep-sequencing experiments in which the primary and relapsed tumors were sequenced at 326× and 270×, respectively. The six benchmark clusters are numbered from C1 to C6 and are represented by shaded ellipses. Radiuses of an ellipse correspond to two standard deviations of VAFs of variants belonging to a benchmark cluster. Dots of the same color are variants assigned to the same cluster by each method. Both MAGOS and PyClone split C5 into two clusters: C5a containing a *TP53* variants and C5b containing a *KRT1* variants. (*D*–*F*) Scatter plots show VAFs of variants from standard depth sequencing experiments in which the primary and relapsed tumors were sequenced at 42× and 47×, respectively.

To represent deep-sequencing scenario, we paired the Illumina WGS data of the primary tumor with the Illumina capture data of the relapsed tumor (median depth = 326× and 270×, respectively). We applied MAGOS, PyClone, and SciClone to this data set and compared the results to the benchmark clusters. All three methods achieved excellent *J*-scores, with the highest score reported by MAGOS (0.94). PyClone that is designed for analyzing multi-sample deep-sequencing data had a *J*-score of 0.92. SciClone had a slightly lower *J*-score (0.89) than the other two methods. Interestingly, no methods inferred six clusters. MAGOS and PyClone both split the benchmark cluster C5 into two (C5a and C5b, fig. 6*A* and *B*). In the Griffith et al. study, 11 variants were tracked over multiple time points, which included a *TP53* variant in cluster C5a and a *KRT1* variant in cluster C5b. VAFs of these two variants diverged over time (fig. 3*A* in Griffith et al. study), supporting that they indeed belonged to distinct subclones, which was consistent with MAGOS and PyClone partitions. PyClone also separated a subset of variants from C2 to form a new group. Conversely, SciClone merged C4 and C6, reporting one less cluster (fig. 6*C*). Temporal tracking of variants in these clusters did not provide sufficient data to evaluate these partitions.

To represent standard depth scenario, we used Ion Torrent capture data for both the primary tumor and the relapsed tumor (median depth = 42× and 47×, respectively). At this reduced depth, VAF distributions of the benchmark clusters had large overlaps. Even for this challenging data set, MAGOS inferred the correct number of clusters, producing a *J*-score of 0.82 (fig. 6*D*). In particular, MAGOS was able to identify the C5 and C6 clusters that had very similar mean VAF in the primary tumor ($\Delta \bar{v}$ = 0.03) and in the relapsed tumor ($\Delta \bar{v}$ = 0.06). PyClone reported 17 clusters (fig. 6*E*). However, because the excessive clusters contained only a few variants, the overall *J*-score was the same as that of MAGOS. SciClone had difficulties of separating overlapping clusters and found only three clusters (*J*-score = 0.78, fig. 6*F*).

These results showed that MAGOS clustering results matched the benchmark clusters the best among the three methods. Furthermore, the reproducibility of MAGOS at different sequencing depths was the highest among the three methods.

### Performance on a Genome with Mixed CNVs and SNVs

Nik-Zainal et al. (2012) performed WGS of a breast cancer sample PD4120a containing somatic SNVs and CNVs. They reconstructed the subclone structure of this tumor via semi-manual analysis and in-depth curations. This data set consisted of 68,749 somatic SNVs and 311 somatic CNVs, which we retrieved via the ICGC data portal (EGAD00001000138; Zhang et al. 2019). Among these variants, 56,974 SNVs were in CN-neutral regions, and the remaining11,775 SNVs were in CNV-affected regions. We used this data set to assess how well MAGOS, PyClone, and SciClone could reproduce the results.

**Fig. 7.**
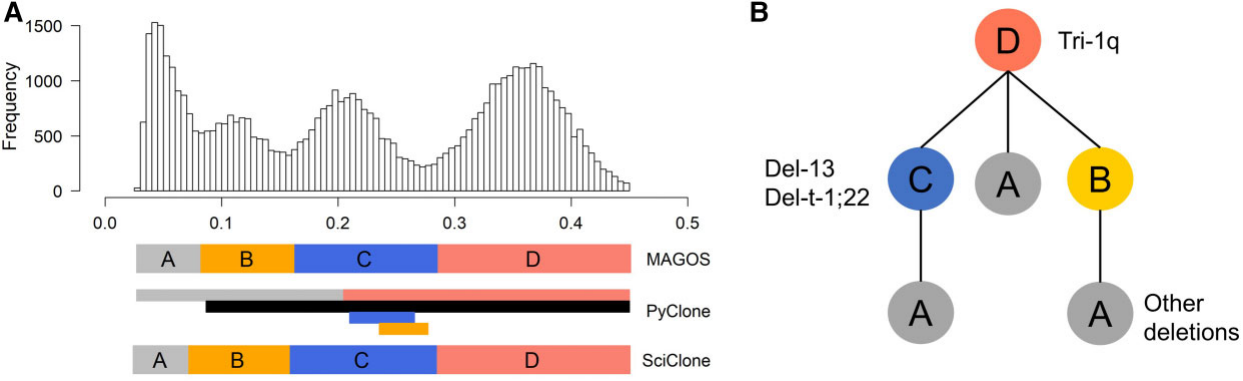
Analysis of the breast cancer sample PD4120a containing mixed SNVs and CNVs. (*A*) Histogram of VAFs of CN-neutral SNVs. Colored bars below the histogram represent clusters identified by each method. Clusters A–D correspond to the four clusters curated by Nik-Zaina et al. (*B*) Phylogenetic relationship of the four clusters resolved by Nik-Zaina et al. Major CNV events in each cluster are displayed. Cluster A likely consists of several subclones, which have not been resolved.

MAGOS and SciClone clustered the SNVs into four subclones, which was consistent with the curated structure (fig. 7*A*). PyClone produced five overlapping clusters that lacked correspondence to the curated structure. Among the three methods, MAGOS is the only one capable of delineating subclone relationship for CNVs and CNV-affected SNVs. Nik-Zaina et al. resolved the evolutionary timing of several major CNV events (fig. 7*B*), including clonal trisomy of chromosome 1 long arm (Tri-1q in cluster D), early subclonal deletion to produce hemizygous chromosome 13 (Del-13 in cluster C), early subclonal deletion to produce a hemizygous translocation between chromosome 1 and chromosome 22 (Del-t:1; 22 in cluster C), and 19 late subclonal homozygous deletions of segments in 11 other chromosomes (in cluster A). MAGOS correctly assigned all clonal and early subclonal events (i.e., Tri-1q in cluster D, Del-13 in cluster C, and Del-t:1; 22 in cluster C). Furthermore, Nik-Zaina et al. identified seven SNVs acquired before Tri-1q because they affected two copies of 1q, which MAGOS recovered perfectly by inferring their affected copy number to be 2 and assigning them to cluster D. MAGOS also inferred the correct subclone membership for seven (36.8%) of the late subclonal events (deletions in cluster A). The misassigned late subclonal events often affected short genomic regions (median length = 68.9 kbps, median SNVs involved = 2). Contrarily, the correctly assigned late subclonal events affected large genomic regions (median length = 500 kbps, median SNVs involved = 7).

These results showed that MAGOS had high accuracy of inferring clusters from mixed SNVs and CNVs and resolving cluster assignments of CNVs affecting large genomic regions.

### Subclone Count as a Prognostic Marker for Late-Stage Liver Cancer

**Fig. 8.**
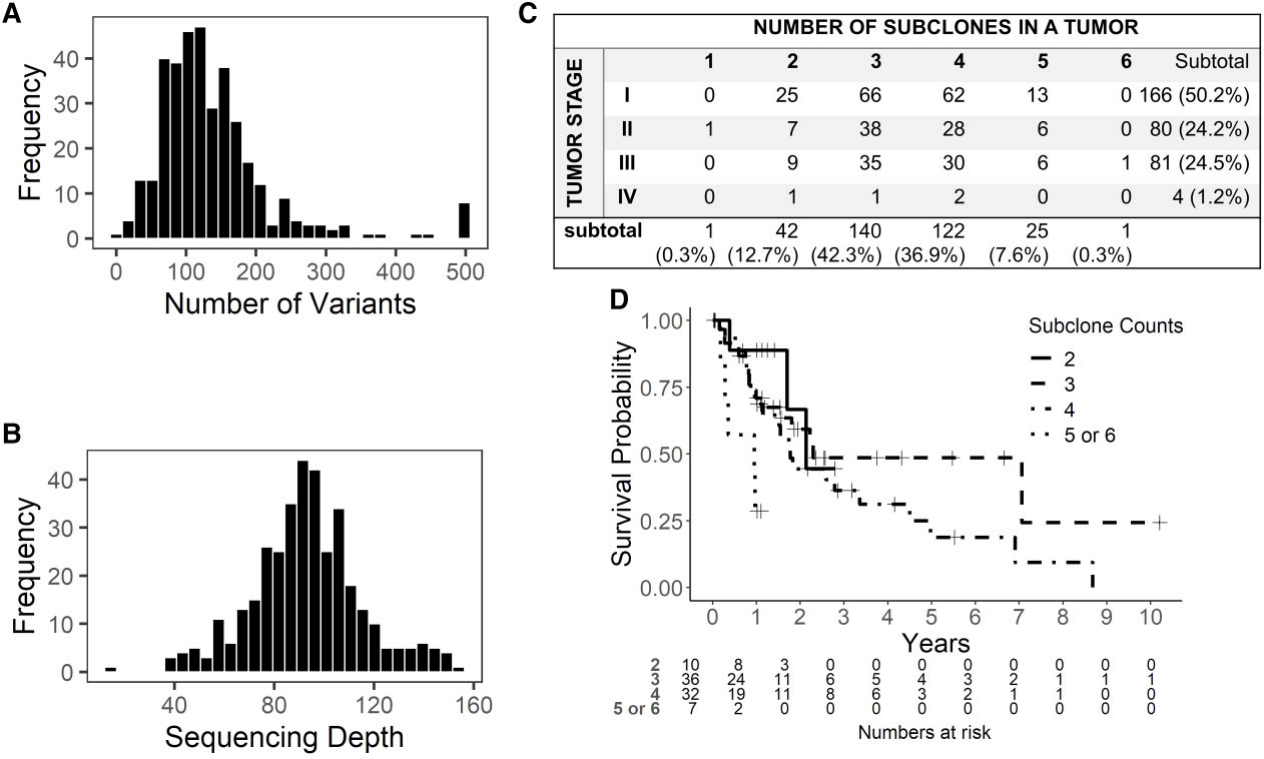
Analysis of TCGA liver cancers. (*A*) Histogram of number of somatic variants in each tumor sample. (*B*) Histogram of median sequencing depth of each tumor sample. (*C*) Summary of liver cancer samples by tumor stage and number of subclones. (*D*) Kaplan–Meier plot of late-stage (stages III and IV) liver cancers. Tumors are stratified into groups based on the number of subclones.

The TCGA project published WESs and clinical data of 331 primary liver hepatocellular carcinomas (TCGA Research Network 2017). On average, each tumor contained 149 somatic variants (fig. 8*A*) and was sequenced at the median depth of 93× (fig. 8*B*).

We applied MAGOS to infer subclonal composition of each tumor. The results showed that a majority (79%) of these tumors contained three to four subclones (fig. 8*C*). To test if age at diagnosis was associated with the number of subclones or the number of variants in a tumor, we built a linear regression model with patient sex, tumor stage, and tumor purity as covariates. This model returned insignificant *P*-values for both subclone count and variant count (*P* = 0.22 and 0.24, respectively). We then tested if patient overall survival was associated with the number of subclones or the number of variants in a tumor. We built a Cox regression model for the 85 late-stage (stages III and IV) tumors using patient sex, age at diagnosis, and tumor purity as covariates. This model showed that high subclone count was a significant risk factor of poor diagnosis (hazard ratio = 1.81, *P* = 0.02; fig. 8*D*), but variant count was not (*P* = 0.74). Contrarily, the model built for the 246 early stage (stages I and II) tumors showed neither subclone counts nor variant counts were associated with overall survival (*P* = 0.96 and 0.07, respectively). These results implied that subclone count was a novel prognostic factor for late-stage liver cancers.

For comparisons, we also applied SciClone and PyClone to these data. Analysis using subclone counts inferred by these methods did not find significant associations with age at diagnosis or overall survival (all *P* > 0.2).

## Discussion

Cancer, as an evolutionary process, has an inherently heterogeneous and dynamic nature (Nowell 1976; Aktipis et al. 2011; Greaves and Maley 2012). Precision identification and intervention of cancers should consider the past, present, and future of each tumor. With high-throughput sequencing technologies, we can now catch snapshots of this process and potentially reconstruct the evolutionary history and trajectory of a tumor (Campbell et al. 2008; Ding et al. 2012; Egan et al. 2012; Nik-Zainal et al. 2012; Welch et al. 2012; Landau et al. 2013). Although single-cell genome sequencing is on the rise, bulk sequencing remains the dominant technology. Bulk sequencing interrogates the amalgam of genomes of heterogeneous cells in the tumor sample and relies on computational analysis to deconvolute the mixed populations.

In this study, we developed a new MAGOS method that infers subclones from bulk sequencing data. By comparing MAGOS, PyClone, and SciClone on simulated and empirical data, we found different strengths and weaknesses of these methods. SciClone requires deep-sequencing data. If the sequencing depth is lower than 300×, the results have low reproducibility and accuracy. Sequencing additional samples of the same tumor does not compensate insufficient sequencing depth for SciClone, as demonstrated in the analysis of the primary-relapsed tumor pair (fig. 6*F*). Conversely, PyClone prefers multiple samples from the same tumor. If only a single-tumor sample is available, increasing sequencing depth does not help the decomposition (fig. 3*D*). PyClone also tends to report excessive small clusters that need to be manually examined (figs. 3*A, B* and 6*E*). A plausible reason is the Dirichlet process in the PyClone algorithm that often overestimates the number of source components (Onogi et al. 2011; Miller and Harrison 2013). Compared with these two methods, the advantages of MAGOS are the most prominent when the sequencing depth is lower than 200×. Even when the sequencing depth drops to 30×, MAGOS can still make accurate cluster inference (*J*-score >0.8), although the $\Delta \bar{v}$ of adjacent clusters needs to be at least 0.25 in single-sample analysis. Adding additional samples from the same tumor improves MAGOS performance significantly: at sequencing depth of ∼40×, MAGOS can decompose clusters with $\Delta \bar{v}$ as low as 0.02–0.06 (fig. 6*D*). Its fast execution also enables subclonal analysis in large cohorts. For example, we applied MAGOS to 331 liver cancers from the TCGA project and discovered subclone counts as a novel prognostic marker.

MAGOS achieved these improvements by modeling the depth-variance and mean-variance dependencies, which reduced the unexplained variance in cluster analysis. It is noteworthy that mean VAF and sequencing depth are not correlated. Increasing sequencing depth has little impact on the centroid of each cluster (figs. 1*B* and 3*A, B*), thus helps little for deconvoluting subclones with similar VAFs. Contrarily, including additional samples from the same tumor helps segregate these clusters that are otherwise undiscernible. Both MAGOS and PyClone show significant performance gain from analyzing multiple samples, suggesting spatial or temporal resampling the same tumor is more cost-effective and robust for subclone characterization than increasing sequencing depth of a single sample. However, sufficient sequencing depth shall be maintained to account for tumor purity, cellular prevalence, and sequencing error that vary from samples to samples (Carter et al. 2012). A good balance between sequencing depth and resampling adjusted for each tumor will be the most beneficial to subclone analysis. Meanwhile, single-cell genome sequencing shows great promise to trace cellular ancestry in a tumor (Kuipers et al. 2017). However, the error models in single-cell data are drastically from that in bulk sequencing data (Zafar et al. 2016). Because MAGOS is based on error models detected in bulk sequencing data, it is inappropriate to apply it to single-cell data. Algorithms that incorporate unique characteristics of single-cell data are an active research field (Jahn et al. 2016; Zafar et al. 2017).

Subclone decomposition is the first step of comprehensive subclonal analyses. The identified clusters are used for further investigations, such as inferring clonal phylogeny (Strino et al. 2013; El-Kebir et al. 2015; Niknafs et al. 2015). LICHeE (Popic et al. 2015) and Rec-BTP (Hajirasouliha et al. 2014) streamline the analysis by first partitioning variants into subclones and then building an evolutionary tree. Given the sequential procedure, the performance of the subclone decomposition step imposes the upper bound of the accuracy of the phylogeny reconstruction step. AncesTree (El-Kebir et al. 2015), CITUP (Malikic et al. 2015), and PhyloSub (Jiao et al. 2014) take a different strategy that infer subclone composition and phylogeny concurrently such that the accuracy of the two steps are jointly optimized. However, these methods require extremely high sequencing depth (200× to >1,000×) and can analyze only a small number of mutations (<100). PhyloWGS (Deshwar et al. 2015) is a new method that supports joint inference using WES or WGS data. Interestingly, when compared with PyClone and SciClone on subclone decomposition, PhyloWGS outperformed these two methods if regions affected by CNVs were mixed with those unaffected by CNVs. When regions affected by CNVs were excluded from the analysis, these three methods performed similarly. Therefore, the superior performance of PhyloWGS is likely due to its correction of CNVs instead of joint optimization. Furthermore, multiple-tumor samples are required to resolve ambiguities in phylogeny. When only a single sample is available, partial phylogeny will introduce uncertainty to subclone decomposition. Decoupling these two steps is a better solution in these cases. Compared with these methods, MAGOS has clear advantages of analyzing large data sets sequenced at low-to-moderate depth in short computational time, clustering SNVs and CNVs, and accommodating single and multiple samples.

MAGOS, as well as PyClone and SciClone, relies on a large number of diploid passenger SNVs that hitchhike with driver mutations in each subclone to detect clusters. When this requirement is not satisfied, such as in ovarian cancers with pervasive CNVs, MAGOS risks missing subclones that predominantly contain CNVs. Therefore, we calculate the fraction of diploid SNVs among all SNVs in a sample and in each subclone and display a warning if this fraction is <0.5. Clusters with low VAFs also need special attention because they may contain neutral variants that are not monophyletic, such as cluster A in figure 7, which may mislead downstream phylogenetic inference (Caravagna et al. 2020). When VAF distributions of subclones overlap, cluster borders reported by computational methods are artificial, and variants close to the borders may belong to neighboring clusters (supplementary material figs. S1 and S2, Supplementary material online). We expect this information can facilitate the optimization of downstream subclonal analysis. Furthermore, when analyzing multiple samples, MAGOS includes all variants including those found in only one sample to increase the power to detect clusters, especially when the sequencing depth is low (supplementary materials and fig. S3, Supplementary material online).

In summary, subclonal diversity provides the fuel for natural selection in neoplasms. It is likely to be robust biomarker for risk stratification, prediction, and prognosis (Maley et al. 2017). MAGOS and future improvements of subclonal decomposition will facilitate basic research and clinical management of cancer.

## Materials and Methods

### Testing Depth-Variance and Mean-Variance Dependence

We used the six benchmark clusters identified by Griffith et al. (2015) in a primary acute myeloid leukemia tumor. For cluster *i* in sequencing experiment *j*, we denote its log-transformed within-cluster VAF as *V*_*i*,*j*_ , its log-transformed median sequencing depth as *D*_*i*,*j*_, its mean VAF as *F*_*i*,*j*_, and its cluster ID as *C*_*i*_. To test if variance of VAF was associated with sequencing depth and mean VAF, we built a mixed-effects model $V_{i,j}\;∼\;\beta D_{i,j}\;+\;\gamma F_{i,j}+A(1\;|\;C_{i})$, in which *β* represents the fixed effect of sequencing depth, *γ* represents the fixed effect of mean VAF, and *A* is a vector of estimated random effects of clusters. We used the R/nlme package (Pinheiro et al. 2017) for this analysis.

### Simulating Subclones in Single-Tumor Samples

To simulate subclones in a single-tumor sample, we used a set of SNVs published by Griffith et al. (2015) which performed WGS data of a primary acute myeloid leukemia tumor. Among 1,343 high-quality validated SNVs, 911 variants were in the founding clone that had a mean VAF of 0.45 (i.e., the estimated purity of the tumor is 90.7%). Using these SNVs as a pool, we created subclones by selecting sets of random variants. For each set, we reduced the mean VAF $\bar{v}$ and the mean sequencing depth $\bar{e}$ by down-sampling the read counts of each allele based on Poisson distributions. Specifically, to create a subclone containing *m* variants with a mean VAF *v*, we first draw *m* random variants from the pool. For a given variant, there are $e_{r}^{0}$ number of reads mapped to the reference allele and $e_{a}^{0}$ number of reads mapped to the alternative allele in the pool. We down-sample these reads to the lower sequencing depth *e* according to Poisson distributions

(8) $$\{\begin{array}{c} e_{r}=\mathrm{Pois}\left(e_{r}^{0}\eta \frac{1-v}{1-u}\right)\\ e_{a}=\mathrm{Pois}\left(e_{a}^{0}\eta \frac{v}{u}\right) \end{array}$$

where $\;\eta =e/(e_{r}^{0}+e_{a}^{0})$, and *e*_*r*_ and *e*_*a*_ are the number of reads mapped to the reference allele and to the alternative allele in the simulated tumor sample, respectively. Using this strategy, we generate two subclones each containing *m* = 100 somatic variants and combine them to create an admixture, representing a single-tumor sample with a two-subclone structure.

### Simulating Subclones in Multiple-Tumor Samples

We used the simulation program published by El-Kebir et al. (2015). This program allows users to specify the number of subclones in a tumor, the number of variants in each subclone, the number of samples sequenced and the average sequencing depth. It then generates random values for cellular prevalence of the subclones and produces read counts for each variant in each sample. We configured each tumor to have three subclones distributed among two, three, or four samples. We set the average sequencing depths to be 30×, 50×, 100×, and 300×. For each combination of sample numbers and sequencing depths, we created 40 simulations.

### Execution of SciClone and PyClone

We downloaded the latest version of SciClone program (v1.1.0) and the PyClone program (v0.13.1) from GitHub. We executed SciClone with these parameters “–do-clustering –maximum-clusters = 10 –minimum-depth = 1 –overlay-clusters.” We executed PyClone by specifying “–density pyclone_binomial” and setting “–tumor_contents” to the true tumor purity value (i.e., 0.9 for simulated data, 0.91 for primary leukemia tumor, and 0.36 for relapsed leukemia tumor) or MAGOS estimated tumor purity values in liver cancer analysis. For CNV data, PyClone did not finish the analysis within 7 days, even using the recommended configuration to speed up the analysis (–max_clusters 10 –init_method connected). We then applied PyClone to 5,000 randomly selected SNVs and reported the result.

### Analyzing Mixed SNVs and CNVs in a Breast Cancer Tumor

Using the International Cancer Genome Consortium data portal (Zhang et al. 2019), we downloaded WGS data published by Nik-Zainal et al. (2012; EGAD00001000138) in VCF format and extracted somatic variant calls in tumor PD4120a. The broad-mutect-v3 file contained 56,615 SNVs with a “PASS” filter and a sequencing depth between 20× and 500×. For each SNV, we retrieved the genomic coordinate and read counts of reference and mutant alleles. The dkfz-copyNumberEstimation file contained 695 CN non-neutral segments and reported total copy numbers and allele-specific copy numbers. After merging overlapping regions, we obtained 311 CNVs. We applied MAGOS with the default settings for single-sample analysis of mixed SNVs and CNV data.

### Preprocessing and Survival Analysis of TCGA Data

Using the Genomic Data Commons data portal (TCGA Research Network 2017), we retrieved clinical data and somatic mutations of 375 liver hepatocellular carcinomas. We used somatic mutations called by the GATK/MuTect pipeline against the hg38 human reference genome. Among these tumors, 331 are primary tumors with clinical information available on age at diagnosis, tumor stage, and overall survival (censored and uncensored). None of these tumors are from the same patient. We applied MAGOS to each tumor and recorded the number of subclones. We grouped tumors into an early stage group (stage I or II) and a late-stage group (stage III or IV). For early stage tumors and for late-stage tumors separately, we tested the association of subclone counts to patient overall survival using Cox proportional hazards regression in which sex and age at diagnosis were covariates. A *P*-value <0.05 indicates significant association.

### Optimizing MAGOS to Improve Computational Efficiency

The standard hierarchical agglomerative clustering procedure requires calculations of all pairwise distances at each step, which leads to an exponential increase of computational complexity as the number of variants grows. Given the narrow range of VAFs between 0 and 1, not all pairwise comparisons are necessary, especially for variants with highly similar VAFs across all samples. To find these variants, we first compute the Euclidean distance between each pair of variants based on their VAFs in all samples. We then construct an incidence matrix and set the entry in row *x* and column *y* to 1 if the Euclidean distance between variants *x* and *y* is <0.01. Using an undirected graph created from this incidence matrix, we search for complete graphs and collapse variants belonging to each complete graph into a leaf node in our initial hierarchical clustering step. Because the number of leaf nodes determines the computational complexity of a bifurcating tree, this step accelerates the speed of MAGOS significantly.

## Supplementary Material

Supplementary data are available at *Molecular Biology and Evolution* online.

## Supplementary Material

msac136_Supplementary_Data

## Data Availability

Data used in this study are available through the ICGC and GDC data portals (EGAD00001000138 and TCGA-LIHC).
