# Supplementary material for: Accurate Identification of Subclones in Tumor Genomes
Source: Mol Biol Evol. 2022 Jun 24;39(7):msac136. doi: 10.1093/molbev/msac136 (PMC9260306; doi:10.1093/molbev/msac136)

**SUPPLEMENTARY MATERIALS**

*J* score

The *J* score is similar to the *%G_E_* value used by Miura et al. who evaluated subclone discovery methods [1]. However, the *J* score accommodates subclone sizes (i.e. number of variants in each subclone) when quantifying the similarities between two sets of clusters, which is based on a commonly used cluster evaluation F-measure [2]. Specifically, we denote $T$ as a set of clusters representing the ground truth, and $D$ as a set of clusters representing predictions. For each cluster $t\in T$, we find its most similar cluster $d\in D$ that has the highest Jaccard index

$$I_{t,d}=\frac{\left| V_{t}\cap V_{d} \right|}{\left| V_{t}\cup V_{d} \right|}$$

where $V_{t}$ is the set of variants belonging to cluster $t$, and $V_{d}$ is the set of variants belonging to cluster $d$. After all truth clusters are matched, if there remain unmatched clusters in $D$, we use the Jaccard index to find their most similar clusters in $T$. For a pair of true cluster $t$ and its best matched predicted cluster $d'$, the clustering recall is $R\left( t \right)=I_{t,d'}$. The overall recall $O=\sum_{t\in T} {(\frac{|V_{t}|}{N}I}_{t,d^{'}})$, where $N$ is the total number of variants analyzed. Similarly, for a pair of predicted cluster $d$ and its best matched true cluster $t'$, the clustering precision is $P\left( d \right)=I_{d,t'}$. The overall precision $PO=\sum_{d\in D} {(\frac{|V_{d}|}{N}I}_{d,t^{'}})$. We then define the J score as

$$J=\frac{2\times RO\times PO}{RO+PO}$$

A *J* score takes a value between 0 and 1, in which 0 means no overlap between any truth clusters and any predicted clusters, and 1 means perfect matches between truth and predictions.

Comparison between using all variants and using only variants shared by all samples

When analyzing multiple samples from the same patient, MAGOS uses both overlapping variants (i.e., those found in all samples) and non-overlapping variants (i.e. those found in a subset of samples) to increase the power to detect clusters, especially when the sequencing depth is low. Eq. 5 in the main text states that between-cluster distance is first computed for each sample separately, then the longest distance is taken as the final value. To illustrates how non-overlapping variants affect sample-specific and cross-sample distances, we applied MAGOS to the paired primary and relapsed tumor data from the Griffith et al. study (Supplementary Figure S3). In general, including non-overlapping variants increases the between-cluster distance in the sample where these variants are unique to (e.g., C1-vs-C2 distance in the relapsed tumor in Fig. S3A) as compared to removing these variants (Fig. S3B). If such increase surpasses the distances in other samples, it will be used as the cross-sample distance, which may lead to the formation of new clusters. We found these cases were common in samples sequenced at the depth around 40x. In this case, including non-overlapping variants correctly identified 4 clusters that were missed by using only overlapping variants (Fig. S3A-B). Contrarily, if such increase does not surpass the longest distance in other samples, it will have no impact. These cases were common in deep-sequenced samples, where including or excluding non-overlapping produced very similar clustering patterns (Fig. S3C-D). The only difference we observed in deep-sequenced samples was an extra small cluster (Fig. S3D), which was likely due to a gap created by removing non-overlapping variants.

References:

1. Miura, S., et al., Predicting clone genotypes from tumor bulk sequencing of multiple samples*.* *Bioinformatics*, 2018. **34**(23): p. 4017-4026.

2. Sundar C, Chitradevi M, Geetharamani G. An analysis on the performance of k-means clustering algorithm for cardiotocogram data clustering. *International Journal on Computational Sciences & Applications (IJCSA)*. 2012;2(5):11-20.

**Supplementary Figure S1**. Probability scores for variants in a simulated single-tumor sample with two subclones. The true clusters (A), MAGOS-identified clusters (B), and SciClone identified clusters (C) are shown. For mis-assigned variants, their MAGOS probabilities are low (D) but SciClone probabilities are high (E).


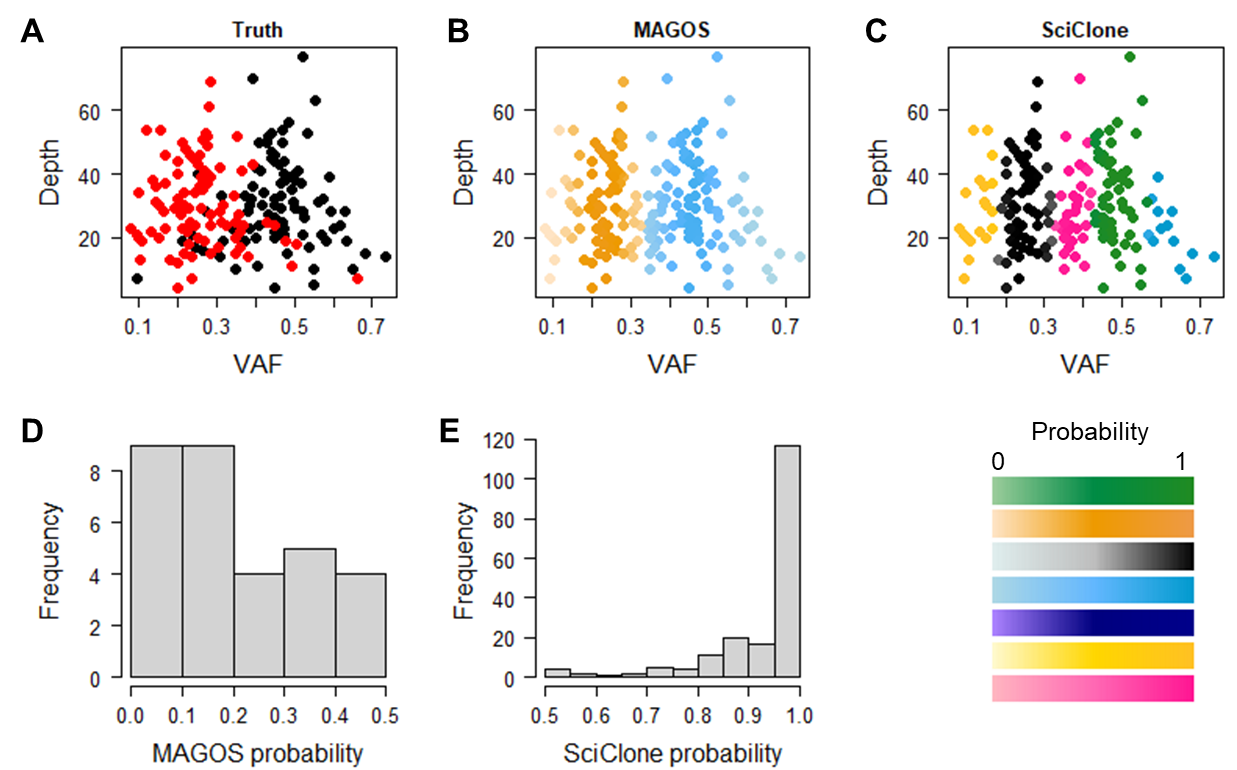


**Supplementary Figure S2**. Probability scores for variants in the empirical data of the paired primary and relapsed tumors. MAGOS probability score are higher for variants close to cluster centers than those at cluster borders (A). SciClone probability scores in general are high and lack variations (B).


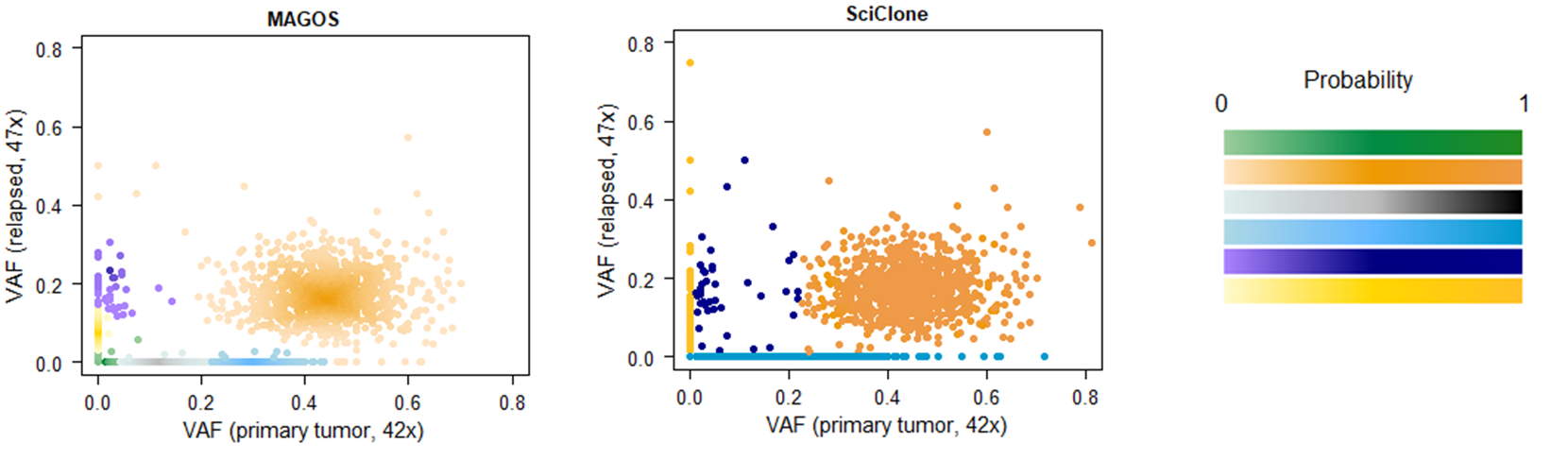


**Supplementary Figure S3**. MAGOS clustering of paired primary/relapsed tumor data from Griffith et al. using all variants (A, C) or using only overlapping variants (B, D). The impact of non-overlapping variants is more significant when sequencing depth is low (~40x, A-B) than when the sequencing depth is high (~300x, C-D). Dots with the same color belong to the same cluster.


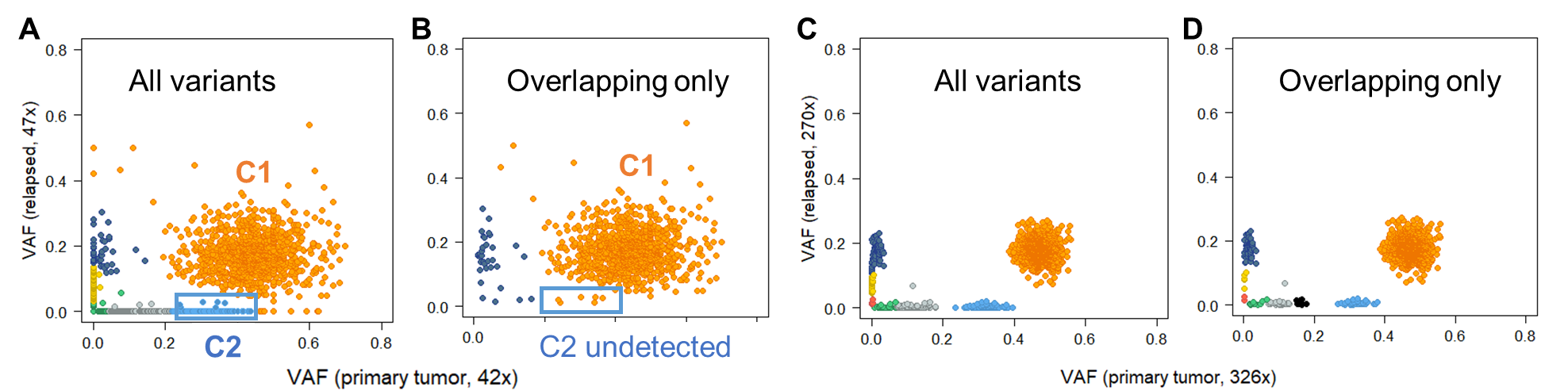

Supplement: msac136_Supplementary_Data [file msac136_supplementary_data.docx]
